# Emory Roybal Center for Dementia Caregiving Mastery: Diverse Responses to Letter of Intent Call

**DOI:** 10.1093/geroni/igaa057.816

**Published:** 2020-12-16

**Authors:** Kenneth Hepburn, Molly Perkins, Drenna Waldrop, Leila Aflatoony, Mi-Kyung Song, Carolyn Clevenger

**Affiliations:** 1 Emory University, Atlanta, Georgia, United States; 2 Georgia Tech, Atlanta, Georgia, United States; 3 Emory University, ATLANTA, Georgia, United States

## Abstract

This new NIA-supported Roybal Center seeks to support Stage 1 pilot clinical trials of programs aimed at promoting caregiving competence and confidence in the great heterogeneity of dementia caregiving contexts. During our first cycle, we received 26 letters of intent (LOI) for full applications. Responses reaffirmed the Center’s premise that dementia caregiving is remarkably varied in nature. While most proposed programs focused on generic caregiving, a number addressed caregiving issues facing specific ethnic/racial groups (African American; Korean American; Native Alaskan/American Indian; Latino), and several focused on specific dementing conditions (MCI, Lewy Body Dementia,TBI-based dementia). Most described programs centered on knowledge development and daily management skill issues (e.g., management of behaviors); others specified development of physical care skills. Decision-making and communication constituted the second most common topic. Over 40% proposed adaptation of existing programs; more than 25% proposed apps or technology interventions. Investigators represented a wide range of disciplines: 45% each from Health sciences (nursing, medicine, and social work) and Social/Behavioral sciences (principally psychology) and the rest from engineering and communications. LOIs varied most in their readiness to complete a clinical trial within a year. About 40% were in very preliminary stages; 25% were clearly poised for a Stage 1 trial; 15% did not sufficiently address the Center’s aims. Key criteria for invitations to submit full applications (n=4) included: specificity of context; clinical trial readiness; reasonableness of proposed adaptation. These criteria should guide future LOIs addressing the diversity of important new research and intervention perspectives on the multifaceted work of caregiving.

